# Genetic polymorphisms associated with sleep-related phenotypes; relationships with individual nocturnal symptoms of insomnia in the HUNT study

**DOI:** 10.1186/s12881-019-0916-6

**Published:** 2019-11-12

**Authors:** Daniela Bragantini, Børge Sivertsen, Philip Gehrman, Stian Lydersen, Ismail Cüneyt Güzey

**Affiliations:** 1 Department of Research and Development (AFFU), Norwegian University of Science and Technology (NTNU), PO Box 3250 Sluppen, NO-7006 Trondheim, Norway; 2 Department of Mental Health, Norwegian University of Science and Technology (NTNU), PO Box 3250 Sluppen, NO-7006 Trondheim, Norway; 3 St. Olav’s University Hospital, Division of Mental Health Care, Østmarkveien 15, NO-7040 Trondheim, Norway; 4 Department of Health Promotion, Norwegian Institute of Public Health, PO Box 973 Sentrum, 5808 Bergen, Norway; 5 Department of Research and Innovation, Helse-Fonna HF Haugesund Hospital, PO Box 2170, 5504 Haugesund, Norway; 6 Department of Psychiatry, University of Pennsylvania Perelman School of Medicine, 3535 Market St., Suite 670, Philadelphia, PA 19104 USA; 7 Regional Centre for Child and Youth Mental Health and Child Welfare (RKBU), Norwegian University of Science and Technology (NTNU), P.O. Box 8905, N-7491 Trondheim, Norway

**Keywords:** Genetics of insomnia, SNPs, Overlapping phenotypes, Sleep traits, The HUNT study

## Abstract

**Background:**

In recent years, several GWAS (genome wide association studies) of sleep-related traits have identified a number of SNPs (single nucleotides polymorphism) but their relationships with symptoms of insomnia are not known. The aim of this study was to investigate whether SNPs, previously reported in association with sleep-related phenotypes, are associated with individual symptoms of insomnia.

**Methods:**

We selected participants from the HUNT study (Norway) who reported at least one symptom of insomnia consisting of sleep onset, maintenance or early morning awakening difficulties, (cases, *N* = 2563) compared to participants who presented no symptoms at all (controls, *N* = 3665). Cases were further divided in seven subgroups according to different combinations of these three symptoms. We used multinomial logistic regressions to test the association among different patterns of symptoms and 59 SNPs identified in past GWAS studies.

**Results:**

Although 16 SNPS were significantly associated (*p* < 0.05) with at least one symptom subgroup, none of the investigated SNPs remained significant after correction for multiple testing using the false discovery rate (FDR) method.

**Conclusions:**

SNPs associated with sleep-related traits do not replicate on any pattern of insomnia symptoms after multiple tests correction. However, correction in this case may be overly conservative.

## Background

In recent years, there has been an increasing focus on the genetic basis for sleep/wake traits. Several genome wide association studies (GWAS) have identified numerous single nucleotide polymorphisms (SNPs) that influence sleep traits [1]. For example, Hu and colleagues reported 15 SNPs, several of which were on circadian genes, that were significantly associated with being a “morning person” [2] while a study by Gottlieb et al. focused on sleep duration identified seven SNPs in two circumscribed genetic loci. Caffeine induced insomnia was the object of another GWAS that identified several loci in melatonin and adenosine pathways [3]. Using the same methodology, Byrne et al. identified several loci with plausible biological role influencing sleep quality and timing [4]. Finally, two studies on the UK biobank sample reported more than a hundred novel SNPs associated to accelerometer registered sleep duration, efficiency and number of nocturnal sleep episodes [5, 6] and reproduced three SNPs from previous studies [6].

These sleep-related traits appear to occur with varying frequencies in individuals with insomnia, a condition characterized by decreased quality and/or quantity of sleep in absence of other organic disorders [7]. A study by Vgontzas et al. reported that individuals with short sleep duration are almost five times more likely to suffer from persistent insomnia [8]. Several studies have shown that individuals with evening chronotype are more likely to present insomnia symptoms [2, 9, 10], in particular difficulties in falling asleep [11]. Therefore, the co-occurrence of specific sleep traits and insomnia might be the product of a common genetic and biological background.

Several GWAS studies seems to support the hypothesis of pleiotropy. A GWAS study by Stein et al. reported an inverted correlation between genetic loci associated with both insomnia and morning chronotype [12]. Several studies conducted on the UK biobank population presented also overlapping genes for insomnia and sleep duration [13–15]. On the other hand, another GWAS study of the same population did not report any common genes for objective measure of several sleep phenotypes [5].

Most studies on insomnia treat insomnia as a single entity and do not consider individual patterns of insomnia symptoms. Insomnia may presents itself as a combination of night-time symptoms but one of these symptom may prevail over the others: difficulties in falling asleep, trouble with staying asleep and waking up too early. Few studies have examined each individual nocturnal symptom of insomnia, despite evidence that the different symptoms may represent biologically distinct mechanisms. Stoffers et al. described decreased gray matter density in a part of the left orbitofrontal cortex in individuals reporting waking up too early, but not in those reporting trouble with falling asleep or sleep maintenance [16]. Epidemiological studies showed that different symptoms are associated with different incidence of physical and psychiatric conditions [17] and mortality [18]. In one such study, males experiencing sleep onset insomnia or terminal insomnia had a risk three-fold higher than healthy sleepers to receive a disability pension due to a mental condition, while maintenance insomnia gave a considerably lower risk.

For these reasons, we argue that investigating individual insomnia symptoms may aid in the identification of genetic overlap with sleep-related traits. Elucidating the relationship between nocturnal insomnia symptoms and sleep-related traits might clarify the etiology and help diagnostic and therapeutic processes.

In order to investigate this relationship, we conducted an association study on individual symptoms of insomnia and SNPs previously reported to be associated with sleep-related phenotypes. The use of material from the Nord-Trøndelag Health Study (HUNT) gave us the opportunity to investigate this relationship in a large sample from a general population.

## Methods

### Participants

This study used data from the Nord-Trøndelag Health Study (HUNT3, Norway) performed in 2006–2008. The study is comprised of 50,807 individuals participated in the study providing extensive health information and biological samples. For a detailed overview of all three HUNT cohorts, see [19].

**Fig. 1:**
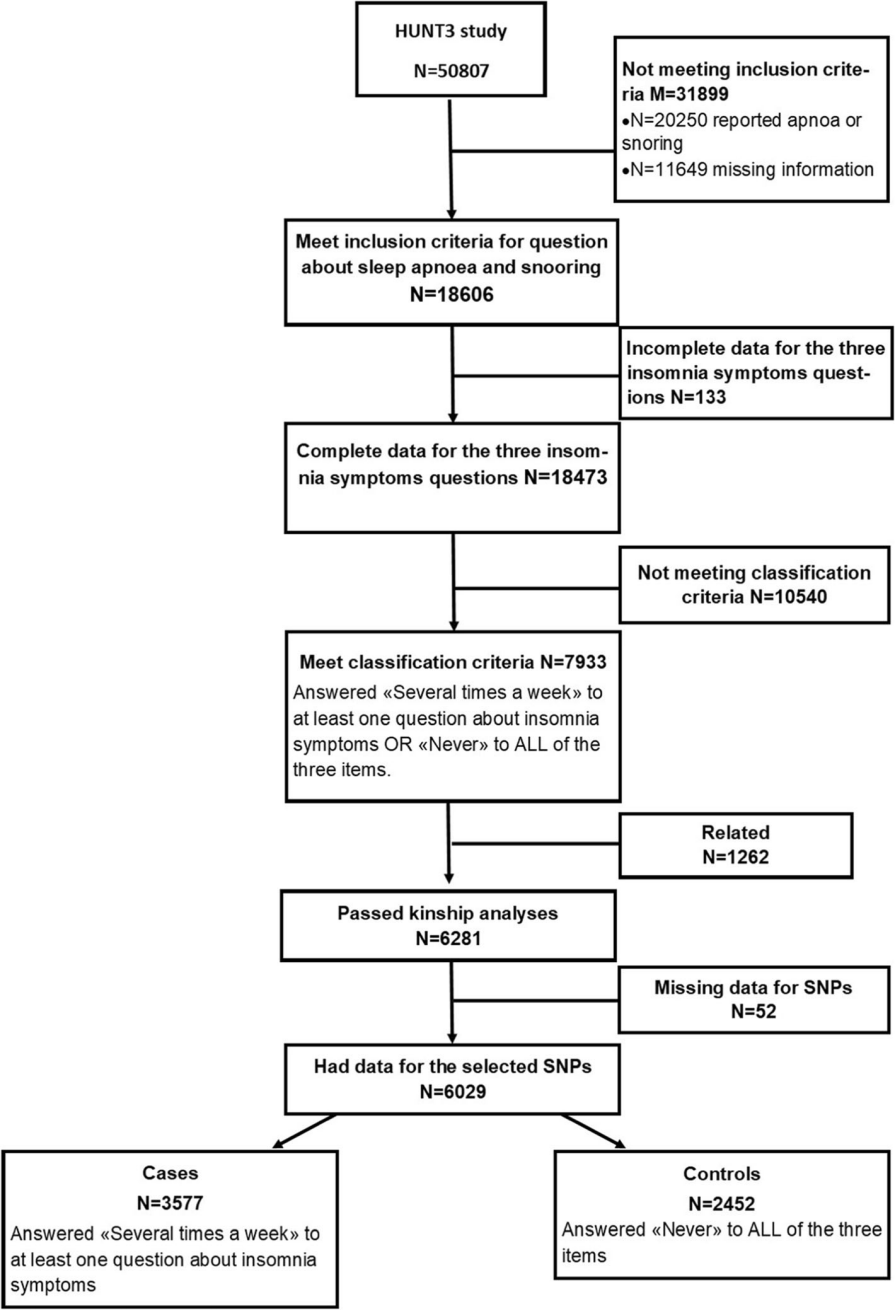
Selection workflow for the HUNT 3 sample included in this study (*N* = 6029)

From the total sample we selected 18,606 participants (36.6%) who answered “Never/Seldom” to questions about snoring and interrupted breathing during the night (individuals answering “Sometimes” and “Several times a week” were excluded). Of these, participants with complete data for symptoms of insomnia (*N* = 18,473, 99.3%) were selected. A total of 7933 participants (43%) could be classified as cases or controls. However, analysis of kinship among these excluded 1262 participants, leaving 6281 participants (79%). Genetic data was available for 6029 of these participants. The selection workflow is shown in detail in Fig. 1.

### Insomnia

To determine the presence of insomnia symptoms we used three questions contained in the HUNT3 Questionnaire 2 (Sleep section [20]). These three questionnaire items inquire about the frequency of the three core symptoms for insomnia disorder, as specified in the Diagnostic and Statistical Manual of Mental Disorders, Fifth Edition (DSM-5) [7].

*“How often in the last 3 months have you:*

*Had difficulty falling asleep at night?*

*Woken up repeatedly during the night?*

*Woken too early and couldn’t get back to sleep?”*

Possible response options were: “Never/seldom”, “Sometimes”, “Several times a week”.

Answering “Several times a week” to at least one question determined cases (*N* = 3577) while answering “Never/Seldom” to all three questions was used as definition for controls (*N* = 2452).

**Fig. 2:**
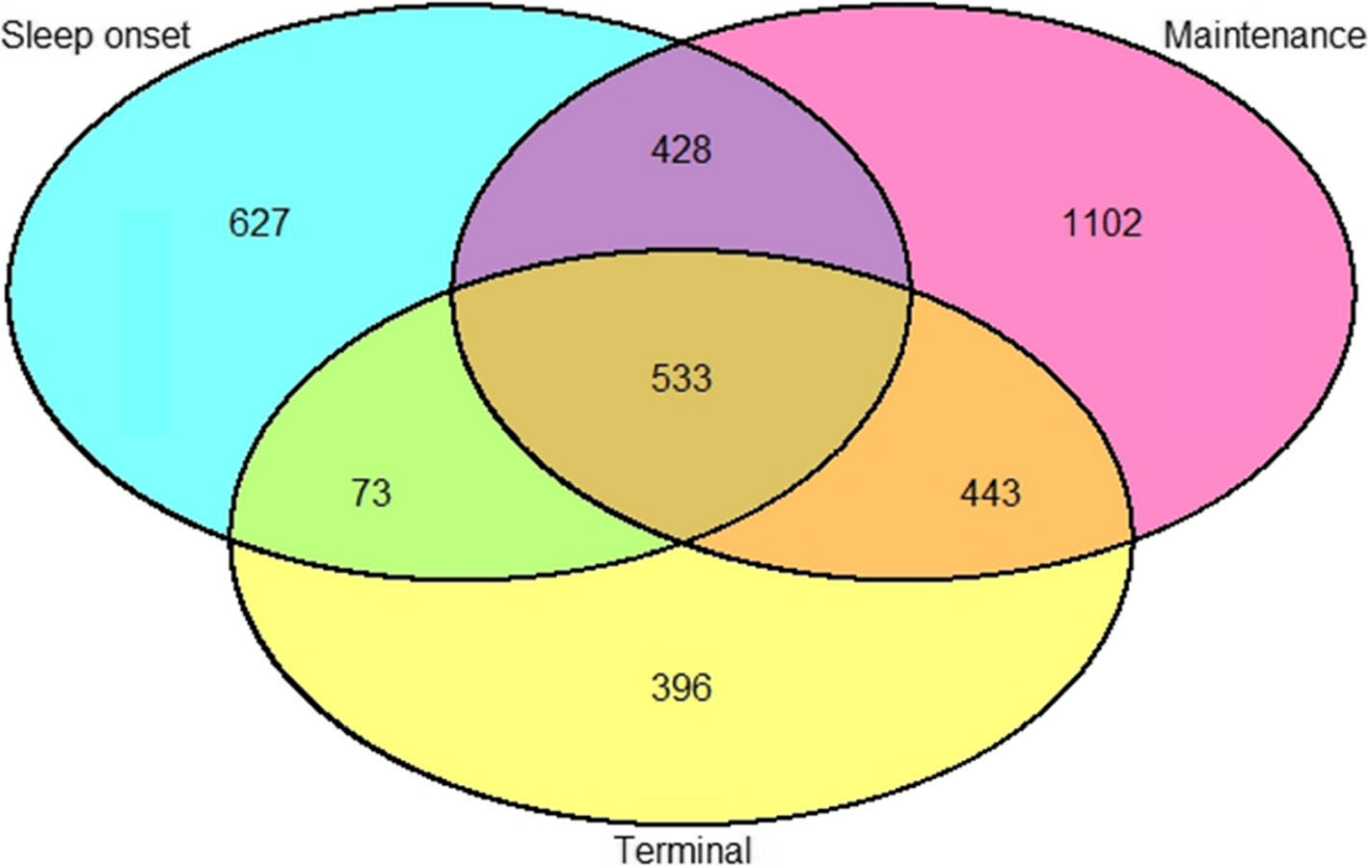
Distribution of the participants according to reported symptoms of insomnia

Cases were further divided in seven subgroups according to the reported pattern of symptoms (Fig. 2).

### Genetic data

We selected the SNPs used in this study the consulting the GWAS catalog [21]. This bioinformatics on-line tool “provides a consistent, searchable, visualizable and freely available database of published SNP-trait associations”. In 2016, we searched the GWAS catalog using the keywords “insomnia”, “sleep” and “chronotype” and selected 52 SNPs from four GWAS studies [3, 4, 22, 23]. Another fifteen SNPs were included from a GWAS study on “morningness” [2] that was not included in the GWAS catalog at the time of the search. A total of 67 SNPs were included in the study.

Genetic data were obtained from the HUNT databank and the genetic material used is stored by the HUNT biobank.

PLINK (version 1.9) [24] was used to exclude SNPs with a minor allele frequency (MAF) below 5% and those not in Hardy-Weinberg equilibrium (*p*-value< 0.05).

We excluded participants who were related up to the third degree (kinship coefficient ≥ 0.0884) using FastIndep [25]. After kinship analysis, 1262 individuals were excluded (98 cases and 1164 controls).

### Analyses

We used multinomial logistic regression to test the association between the 67 SNPs and the traits (patterns of symptoms of insomnia plus controls as a dependent variable with eight categories). Sex and age in years were included as covariates. Correction for multiple statistical hypotheses was conducted using Benjamini-Hochberg False Discovery Rate (FDR) (58 SNPs × 7 comparisons for a total of 413 tests, alpha = 0.05). All the statistical analyses were conducted using RStudio (Version 1.0.136).

## Results

### Descriptive statistics

Descriptive statistics are shown in Table 1. The current sample (*N* = 6029) included more females (67%) than males. Mean age for the whole sample was 50, (SD = 16.2, range: 19.2 to 96.8 years), 53 for cases and 45 for controls (t (5545,3) = − 20.55, *p* > 0.001).

**Table 1:** Descriptive statistics for the sample

	Count	Females (%)	Age (M)
Sleep onset (SOI)	627	71.1	48.9
Maintenance (MI)	1102	72.5	53.0
Terminal (TI)	396	60.6	57.6
SOI + MI	428	80.6	52.1
MI + TI	443	68.6	55.6
SOI + TI	73	78.1	58.7
SOI + MI + TI	533	83.1	55.1
Total in cases	3602	73.1*	53.4*
Controls	2427	58.2	45.2
Total	6029	67.1	50.1

* = *p* < 0.05 for comparison of cases versus controls

### Association testing

Nine SNPs with MAF lower than 5% in the CEU population (Northern Europeans from Utah) or our sample were excluded. Therefore, a total of 58 SNP were tested for associations.

Sixteen SNPs presented *p*-values below 0.05 before correction for multiple testing in eighteen associations (two SNPs were associated with two symptoms sub-groups). None of the p-values retained significance after Benjamini-Hochberg FDR correction.

Besides the p-value, the odds ratio for most of the tests could be considered valid. The T allele of rs2302729 on *CACNA1C* (Calcium voltage-gated channel subunit alpha1 C) were 1.9 times more likely than controls (*p* = 0.004, 95% CI [1.2 to 3]) the highest odds ratio for experiencing sleep onset insomnia with terminal insomnia. For the T allele of rs10493596 on gene *AK5* (Adenylate Kinase 5) the same symptom was 66% less likely to occur (*p* = 0.004, OR = 0.4, 95%CI [0.3 to 0.8]) compared to controls. Individuals who reported sleep onset insomnia with maintenance insomnia were 50% more likely than controls to present the T allele of rs113851554 on *MEIS1* (*p* = 0.01, OR = 1.5, 95% CI [1.1 to 2]). Annotations for all 16 SNPs are collected in Table 2. Results for all SNPs are presented in Additional file 1.

**Table 2:** SNPs showing significant p-value before FDR correction

SNP	Ref. allele	Other allele	Gene	Symptoms sub-group	B	OR	95% CI	*p*-value	Previous associations
rs10493596	T	C	*AK5*	SOI + TI	−0.82	0.45	[0.3 to 0.8]	0.004	Morning chronotype [2]
rs10823607	T	C	*ADAMTS14*	SOI + TI	− 0.71	0.5	[0.2 to 0.9]	0.039	Sleep duration [4]
rs113851554	T	G	*MEIS1*	SOI + MI	0.4	1.5	[1.1 to 2]	0.007	Insomnia symptoms [13]
				SOI + MI + TI	0.36	1.4	[1.1 to 2]	0.007	
rs11706236	G	A	*CACNA2D3*	MI + TI	−0.33	0.7	[0.5 to 0.9]	0.009	Caffeine related insomnia [3]
rs12471454	T	C	*SATB2*	TI	−0.26	0.8	[0.6 to 1]	0.029	Insomnia [4]
rs12927162	G	A	*TOX3*	MI	−0.15	0.9	[0.7 to 1]	0.036	Morning chronotype [2]
rs1823125	G	A	*PAX8*	MI	0.16	1.2	[1 to 1.4]	0.033	Sleep duration [23]
rs1940013	T	C	*OPCML*	SOI	0.19	1.2	[1 to 1.5]	0.037	Usual bedtime [22]
rs2221285	T	C	*ESRRG*	SOI + TI	−0.53	0.8	[0.7 to 1]	0.027	Sleep duration [23]
rs2287838	G	A	*PIN1*	SOI	−0.22	0.8	[0.7 to 1]	0.022	Sleep duration [23]
rs2302729	T	C	*CACNA1C*	SOI + TI	0.64	1.9	[1.2 to 3]	0.009	Sleep latency [4]
rs34714364	T	G	*APH1A*	MI	0.16	1.18	[1 to 1.4]	0.041	Morning chronotype [2]
rs55694368	T	G	*PER2*	MI	−0.19	0.83	[0.7 to 1]	0.043	Morning chronotype [2]
rs6437122	G	C	*UPP2*	SOI + MI + TI	−0.34	0.7	[0.5 to 0.9]	0.015	Sleep duration [23]
rs9517132	T	C	*RANBP5*	SOI	−0.22	0.8	[0.7 to 1]	0.018	Usual sleep duration [23]
rs9804200	C	T	*EBF3*	MI + TI	−0.21	0.81	[0.7 to 1]	0.044	Usual bedtime [4]

*SOI* sleep onset insomnia, *MI* maintenance insomnia, *TI* terminal insomnia

## Discussion

Sixteen SNPs previously associated with sleep-related traits were significantly associated with at least one symptom or a combination of symptoms of insomnia. However, none of these variations stayed significant after correction for multiple statistical testing.

Among our highest hits, there was SNP rs10493596. This variation is close to the *AK5* (Adenylate Kinase 5) gene that encodes for an adenylate kinase expressed exclusively in the brain. This protein is involved in ATP homeostasis by catalyzing the transfer of phosphate groups among adenine nucleotides. Rs10493596 was associated with “morningness” in a study by Hu et al. [2] while in our study it gave the lowest *p*-value for difficulties falling asleep in combination with early morning awakenings.

Difficulties in falling asleep with early morning awakenings showed also an association with rs2302729 that was previously associated with sleep quality and latency [4]. This SNP is located on *CACNA1C* (Calcium Voltage-Gated Channel Subunit Alpha1 C) whose involvement in several psychiatric conditions is supported by epidemiological and animal studies [26]. Knockout mice for *CACNA1C* display traits that resemble symptoms of mental disorders and autism such as cognitive decline, anxiety, hyperactivity, decreased sociability, decreased synaptic plasticity [27].

Of note is the presence of a polymorphism on gene *MEIS1* among our highest hits. *MEIS1* is involved in restless leg syndrome (RLS) [28] but recently also reported to be associated with insomnia symptoms [5, 13, 29]. In our study, combinations of symptoms sleep onset problems with maintenance insomnia and all symptoms together showed low *p*-values (0.01 and 0.02 respectively) and discrete odds ratio (1.5 and 1.4) for the T allele of rs113851554, in agreement with previous studies. This finding strengthens the hypothesis that insomnia and RLS may be overlapping phenotypes not easy to discern [13].

The A allele of rs12927162 on gene*TOX3* (TOX High Mobility Group Box Family Member 3), decreased the chances of maintenance insomnia. This SNP is reported in significant association with being a morning person [2] but also with measure of circadian phase delay [5].

Rs1823125 near gene *PAX8* was firstly reported as associated with sleep duration in the CHANGE consortium sample [23], and successively in the UK Biobank sample [6] in which it was associated also with sleep efficiency [5]. In our study, it was associated with maintenance insomnia. PAX8 is a transcription factor with proven role in kidney and thyroid morphogenesis. Its role on sleep is yet to be investigated, and it is possible that rs1823125 is not in fact influencing *PAX8* but another gene nearby as it is located in a intragenic region.

Most of our results seems to have a plausible explanation in spite of ending up statistically non-significant after correction for multiple testing. The need for correction when testing multiple hypotheses may often be redundant especially in the context of biology or in K. J. Rothman words “… when scientists are studying biological relations rather than random numbers, the premise that type I errors are the major concern may be wrong” [30]. Therefore overestimating the role of “chance” when analyzing biological data may lead to type II errors. In our case, we chose SNPs known to influence sleep-related traits, therefore their involvement in insomnia is plausible.

### Strengths and limitations

The HUNT study collected data from Norwegians from the region of Nord-Trøndelag which gives the advantage of a relative-high genetic isolation and exposure to similar environmental factor that may influence sleep (natural light, cultural habits etc.). Also, strong welfare polices implemented in Norway lessen the effect of socioeconomic disparities that may affect the analyses.

Unfortunately, the HUNT study did not gather information about sleep length or satisfaction that could have helped determining the presence of an actual disorder. Inclusion of this information in future studies and the finding of endophenotypes may help the discovery of relevant genetic associations.

Several of our significant SNPs (before correction for multiple testing) were associated with the subgroup of symptoms “sleep onset with early morning awakenings”. This combination of symptoms was the rarest, with only 73 individuals reporting it. This further support the strength of the association.

## Conclusions

After multiple testing correction, we did not find any statistically significant association between combination of symptoms of insomnia and several SNPs associated with sleep-related phenotypes. However, the presence of a biological explanations and early reports on similar phenotypes makes vigorous use of correction for multiple statistical testing questionable.

## Supplementary information

**Additional file 1.** Full results for the multinomial regression analysis.

## Data Availability

Data used in this study is available on request to the HUNT databank (https://hunt-db.medisin.ntnu.no/hunt-db/#/) and is subject to fees as decided by the HUNT Research Centre (https://www.ntnu.edu/hunt/data). The authors of the present study do not have permission to share the dataset obtained from HUNT.

## Abbreviations

*ADAMTS14*: ADAM Metallopeptidase with Thrombospondin Type 1 Motif 14
*AK5*: Adenylate Kinase 5
*APH1A*: Aph-1 Homolog A, Gamma-Secretase Subunit
ATP: Adenosine triphosphate
*CACNA1C*: Calcium voltage-gated channel subunit alpha1 C
*CACNA2D3*: Calcium voltage-gated channel auxiliary subunit alpha 2 Delta 3
CEU: Utah Residents (CEPH) with Northern and Western European Ancestry
DSM-5: Diagnostic and Statistical Manual of Mental Disorders, Fifth Edition
*EBF3*: EBF Transcription Factor 3
*ESRRG*: Estrogen Related Receptor Gamma
FDR: False discovery rate
GWAS: Genome wide association study
HUNT: Helse Undersøkelse Nørd-Trøndelag (Nord-Trøndelag Health Study)
MAF: Minor allele frequency
*MEIS1*: Meis Homeobox 1
*OPCML*: Opioid Binding Protein/Cell Adhesion Molecule Like
*PAX8*: Paired Box 8
*PER2*: Period Circadian Regulator 2
*PIN1*: Peptidylprolyl Cis/Trans Isomerase, NIMA-Interacting 1
*RANBP5*: Uridine Phosphorylase 2
*SATB2*: SATB Homeobox 2
SNPs: Single nucleotide polymorphisms
*TOX3*: TOX High Mobility Group Box Family Member 3
*UPP2*: Uridine Phosphorylase 2
